# Pelargonium Extract EPs 7630 in the Treatment of Human Corona Virus-Associated Acute Respiratory Tract Infections – A Secondary Subgroup-Analysis of an Open-Label, Uncontrolled Clinical Trial

**DOI:** 10.3389/fphar.2021.666546

**Published:** 2021-04-30

**Authors:** Tilman Keck, Andreas Strobl, Andreas Weinhaeusel, Petra Funk, Martin Michaelis

**Affiliations:** ^1^ Department of ENT Medicine, Head and Neck Surgery, Hansa Private Hospital, Graz, Austria; ^2^ Department of ENT Medicine, Head and Neck Surgery, Ordensklinikum Linz, Krankenhaus Barmherzige Schwestern, Linz, Austria; ^3^ Health and Environment Department, Molecular Diagnostics, AIT Austrian Institute of Technology GmbH, Wien, Austria; ^4^ Medical Scientific Services, Dr. Willmar Schwabe GmbH and Co. KG, Karlsruhe, Germany; ^5^ Industrial Biotechnology Centre and School of Biosciences, University of Kent, Canterbury, United Kingdom

**Keywords:** acute respiratory tract infection, common cold, human coronavirus, Pelargonium sidoides, treatment

## Abstract

**Background:** Experience in treating human coronavirus (HCoV) infections might help to identify effective compounds against novel coronaviruses. We therefore performed a secondary subgroup-analysis of data from an open-label, uncontrolled clinical trial published in 2015 investigating the proanthocyanidin-rich Pelargonium sidoides extract EPs 7630 in patients with the common cold.

**Methods:** 120 patients with common cold and at least 2 out of 10 common cold symptoms received one film-coated 20 mg tablet EPs 7630 thrice daily for 10 days in an uncontrolled, interventional multicentre trial (ISRCTN65790556). At baseline, viral nucleic acids were detected by polymerase chain reaction. Common cold-associated symptoms and treatment satisfaction were evaluated after 5 days and at treatment end. Based on the data of patients with proof of viral nucleic acids, we compared the course of the disease in patients with or without HCoV infection.

**Results:** In 61 patients, viral nucleic acids were detected. Of these, 23 (37.7%) were tested positive for at least one HCoV (HCoV subset) and 38 (62.3%) for other viruses only (non-HCoV subset). Patients of both subsets showed a significant improvement of common cold symptoms already after 5 days of treatment, although the observed change tended to be more pronounced in the HCoV subset. At treatment end, more than 80% of patients of both groups were completely recovered or majorly improved. In both subsets, less than 22% of patients took concomitant paracetamol for antipyresis. The mean number of patients’ days off work or school/college was similar (0.9 ± 2.6 days in HCoV subset vs 1.3 ± 2.8 days in non-HCoV subset). In both groups, most patients were satisfied or very satisfied with EPs 7630 treatment.

**Conclusion:** EPs 7630 treatment outcomes of common cold patients with confirmed HCoV infection were as favourable as in patients with other viral infections. As this trial was conducted before the pandemic, there is currently no evidence from clinical trials for the efficacy of EPs 7630 in patients with SARS-CoV-2 infection. Dedicated non-clinical studies and clinical trials are required to elucidate the potential of EPs 7630 in the early treatment of HCoV infections.

## Introduction

The current COVID-19 pandemic has boosted interest in treatment options for human coronavirus (HCoV) infections. Currently, seven HCoVs have been reported (Liu et al., 2020). The novel betacoronaviruses (β-CoVs) MiddleEast respiratory syndrome coronavirus (MERS-CoV), severe acute respiratory syndrome coronavirus (SARS-CoV) and SARS-CoV-2 cause the most severe respiratory syndromes. However, severe and life-threatening complications like pneumonia have also been reported for other HCoVs (Schildgen, 2018; Pillaiyar et al., 2020). Due to the route of infection via the respiratory epithelium, there are similarities in the clinical picture between SARS-CoV-2 and other HCoVs (Rothan & Byrareddy, 2020). At disease onset, cough, sputum production, and sore throat are frequently present. Fever, fatigue, and headache are reported at later phases. Experience from treating infections with common-cold associated HCoVs [i.e., HCoV-HKU1, HCoV-OC43, HCoV-NL63, HCoV-229E (Liu et al., 2020)] might therefore support the identification of compounds with therapeutic potential against novel severe coronavirus infections.

Herbal medicinal products together with NSAIDs, vitamins, and trace elements are commonly used in the treatment of acute upper respiratory tract infections, which are mostly caused by viruses. Among others, active substances include tannins, e.g., proanthocyanidins, which are known to interact with viral proteins and are able to change or inhibit their structure and functionality (Hensel et al., 2020). Particularly, proanthocyanidins exert antiviral effects such as inhibition of viral proteins involved in cell adhesion or virus release (Derksen et al., 2014; Quosdorf et al., 2017; Tsukuda et al., 2017), inhibition of virus-induced autophagy (Dai et al., 2012), and inhibition of host cell signalling (Lee et al., 2017).

The proanthocyanidin-rich extract EPs 7630 (EPs^®^ 7630 is a proprietary extract and active ingredient in pharmaceuticals manufactured by Dr Willmar Schwabe GmbH and Co. KG.) from the roots of Pelargonium sidoides possesses promising immune-modulating as well as antiviral properties (Antonelli et al., 2020; Brendler et al., 2020; Mani et al., 2020; Silveira et al., 2020). Pelargonium sidoides is a plant native to South Africa, which shares the same genus (Pelargonium) with the common ornamental geraniums and which roots have been used as herbal remedies for respiratory and gastrointestinal infections centuries-long by the local South African populations (Careddu & Pettenazzo, 2018). The plant became known in Europe 120 years ago, when it was described by the Englishman Charles Henry Stevens and its root praised as a new remedy for tuberculosis (Bladt & Wagner, 2007). Today, EPs 7630 is the active ingredient of medicinal products in both tablet and liquid forms and used in children from the age of one year, adolescents and adults for the treatment of respiratory tract infections in several countries in Europe, Asia, Australia, as well as Central and South America. In adults, the recommended daily dose is 30 drops of liquid solution or one 20 mg tablet thrice daily.

A number of non-clinical studies demonstrated activity of EPs 7630 against a variety of respiratory viruses such as HCoV (HCo-229E), influenza A virus (H1N1, H3N2), respiratory syncytial virus (RSV), and parainfluenza virus (Michaelis et al., 2011; Theisen & Muller, 2012). In cell culture experiments, EPs 7630 inhibited the attachment of HIV-1 to human immune cells and viral entry (Helfer et al., 2014). Moreover, EPs 7630 inhibited influenza A virus replication by inhibition of hemagglutinin and neuraminidase activity (Theisen & Muller, 2012), which was at least in part mediated by the proanthocyanidin constituents. In human bronchial epithelial cells, EPs 7630 reduced rhinovirus-16 replication by downregulating the expression of inducible co-stimulator (ICOS) and its ligand (ICOSL) as well as the surface calreticulin receptor, whereas levels of host defence supporting proteins were increased (Roth et al., 2019). Mechanistically, EPs 7630 significantly reduced host cell attachment of various viruses and prevented virus release from infected cells. Moreover, EPs 7630 stimulated the release of nitric oxide (NO), type I interferon (IFN), and different cytokines involved in host defense mechanisms (Kolodziej, 2011; Witte et al., 2015; Witte et al., 2020). In athletes, EPs 7630 modulated the immune response during strenuous exercise by increasing immunoglobulin α production in saliva, decreasing interleukin (IL)-15 and IL-6 levels in the serum, and reducing IL-15 in the nasal mucosa (Luna et al., 2011). EPs 7630 was also found to prevent asthma attacks provoked by rhinovirus in children, probably by interfering with IL-6-, IL-8-, and IL-16-mediated inflammation (Tahan & Yaman, 2013).

Over the last 25 years, more than 30 clinical trials have investigated EPs 7630 for the treatment of acute respiratory tract infections (ARTI) (Brendler et al., 2020). Systematic reviews and meta-analyses have provided evidence that EPs 7630 is effective and well-tolerated in adults, children and adolescents with ARTI such as acute bronchitis or the common cold (Agbabiaka et al., 2008; Timmer et al., 2013; Matthys et al., 2016; Kamin et al., 2018; Schapowal et al., 2019; Seifert et al., 2019; Wopker et al., 2020; Seifert et al., 2021). Most randomized controlled trials investigating EPs 7630 were performed in patients with acute bronchitis. A systematic meta-analysis of placebo-controlled clinical trials showed the efficacy of treatment with EPs 7630 in this indication, with a significantly faster and more effective reduction of bronchitis-specific symptoms (Agbabiaka et al., 2008). For the indication common cold, a first double-blind, randomized controlled trial in 103 adults was reported in 2007, in which patients were treated with EPs 7630 (3 × 30 drops/d) or placebo for up to 10 days (Lizogub et al., 2007). Until day 5, the primary outcome measure “Sum of the Symptom Intensity Differences” (SSID) had significantly improved by 14.6 ± 5.3 points (mean ± SD) in the EPs 7630 group compared to 7.6 ± 7.5 points in the placebo group of this trial. The mean duration of inability to work was also significantly lower in the EPs 7630 group compared to placebo. In a second part of this trial, which was published separately (Riley et al., 2018), 104 patients with common cold were randomized to a ten-days treatment with 3 × 60 drops/d EPs 7630 or placebo. Again, the mean SSID from baseline to day 5 was statistically significantly higher for EPs 7630 compared to placebo (16.0 ± 7.4 vs. 8.3 ± 7.6 points). After 10 days, 90.4% of EPs 7630 patients and 21.2% of placebo patients were clinically cured. In another trial reported in 2019 (Riley et al., 2019), 105 adults suffering from common cold symptoms were randomized to a treatment with one film-coated tablet containing 40 mg EPs 7630 or matched placebo thrice-daily for 10 consecutive days. In this trial, the SSID had significantly improved by 12.5 ± 4.4 points the EPs 7630 group compared to 8.8 ± 6.8 points in the placebo group at day 5. Moreover, on day 10, EQ-VAS status, activity level, and the general well-being were significantly better in the EPs 7630 group than in the placebo group.

The favourable safety profile of EPs 7630 has been confirmed in clinical trials with a total of more than 10,000 patients including adults, adolescents and children as well as from decades of market experience (Matthys et al., 2013; Careddu & Pettenazzo, 2018; Brendler et al., 2020).

Recently, we reported on an open-label, interventional, uncontrolled, phase IV clinical trial designed to obtain further information on the tolerability and effectiveness of EPs 7630 in a real-world setting in patients suffering from the common cold (Keck et al., 2015). This clinical trial showed the excellent tolerability of EPs 7630 in a 10 days’ treatment of adult patients suffering from the common cold. Moreover, most patients showed a favourable course of recovery from the disease. In a secondary subgroup-analysis of trial data (Keck et al., 2018), the association between the detection of viral nucleic acids at the start of treatment and the subsequent course of the disease specific symptoms was further investigated. This analysis revealed that the most frequently detected viruses by molecular testing at baseline were enterovirus/rhinovirus followed by HCoV-HKU1 and HCoV-OC43 (Keck et al., 2018).

We performed a secondary subgroup-analysis of this clinical trial and compared treatment outcomes in patients with or without HCoV to investigate whether the proanthocyanidin-rich extract EPs 7630 could be helpful in the treatment of HCoV-associated ARTI.

## Materials and Methods

This was a secondary subgroup-analysis of a 10 days open-label, uncontrolled, interventional multicentre phase IV clinical trial conducted in the out-patient clinics of eight hospitals in Austria. The trial was registered at the ISRCTN registry (ISRCTN65790556) and its protocol was approved by the responsible ethics committee. The principles of Good Clinical Practice and the Declaration of Helsinki were adhered to. All patients provided written informed consent.

Details on the methodology and the main results for the total population of the underlying clinical trial are described elsewhere (Keck et al., 2015; Keck et al., 2018). In the following, only the methodological details and results for the subset analysis are given.

### Participants

Eligible participants were male or female outpatients aged 18 years or older, with a clinical diagnosis of common cold and at least two of the following 10 common cold symptoms (CCS):

• sneezing
• nasal discharge
• nasal congestion
• scratchy throat
• hoarseness
• sore throat
• coughing
• headache
• malaise
• fever

Exclusion criteria were: any acute respiratory tract disease other than the common cold, obstructive anatomic nasopharyngeal lesions (e.g., nasal polyps), severe septal deviations, previous or planned surgery of the nose or paranasal sinuses, chronic pulmonary diseases, allergic rhinitis, conditions known to cause sore throat such as tonsillopharyngitis, drugs, aphthous ulcers, or candida.

### Interventions

Patients took one film-coated tablet EPs 7630 (Kaloba®, Austroplant Arzneimittel, Vienna, Austria) thrice daily for 10 days. Each tablet contained 20 mg herbal drug preparation from the roots of Pelargonium sidoides (1:8–10), dried; extraction solvent: ethanol 11% (w/w). Roots of Pelargonium sidoides were collected in South Africa (e.g., Eastern Cape). The dried material was tested in an array of biochemical and phytochemical methods in order to confirm the quality and identity of the herbal material. Pharmacognosy was done by the quality control department of Dr. Willmar Schwabe GmbH and Co. KG. A voucher specimen of every lot is deposited in the Department of Pharmacognosy to be retained for ten years. In addition, the herbal material is tested with respect to purity (e.g., heavy metals, and microbiological quality). EPs 7630 is classified by the European Pharmacopoeia as “other extract” and therefore not adjusted to a specific content of constituents. Independent of this formal classification, the constituents of this herbal active ingredient are described in detail by Schoetz and colleagues (Schoetz et al., 2008). Approximately 80% m/m of the extract are assigned to six major groups of constituents, with oligomeric prodelphinidines (commonly designated in this context as polyphenols) being the most significant group (approximately 40% of the dried extract). The film-coated tablets used in the underlying clinical trial were tested and released compliant with drug GMP and the European Pharmacopoeia according to written, authorized and validated analytical procedures with respect to identity, extract content, uniformity, disintegration, and microbiological purity.

Treatment compliance was assessed by counting the number of the remaining film-coated tablets at trial end.

Concomitant common cold medication which might impair the interpretation of trial results was not allowed except for up to 500 mg paracetamol every 6 h in the case of fever over 38.5°C.

### Outcomes

At each visit (baseline, day 5, and day 10), the investigator assessed the 10 CCS (see above) on a 4-point rating scale (0 = not present to 3 = severe). Ratings were then summed up to a total CCS score.

Furthermore, the 8 additional common cold-relevant complaints (CRC)

• pulmonary rales at auscultation
• sputum production
• chest pain while coughing
• chills
• exhaustion
• loss of appetite
• diarrhoea
• muscle aches

were assessed by the investigator on a 4-point rating scale (0 = not present to 3 = severe). These 8 individual ratings were summed up to a total CRC score.

An overall symptom score was calculated from the total CCS and CRC scores.

Participants were instructed to record the intensity of 15 disease-related symptoms (runny nose, congested nose, sneezing, sore throat, scratchy throat, hoarseness, coughing, headache, malaise, chills, chest pain during coughing, loss of appetite, restless sleep, limitation of usual daily activities, and muscle ache) by means of a 5-point rating scale (0 = not at all to 4 = severe) in the diary and a total score was calculated.

Moreover, we laid a focus on symptoms of the CCS or CRC described as similar between SARS-CoV-2, earlier β-CoVs as well as other common acute upper respiratory tract infections (fever, cough, malaise, sputum production, headache, and sore throat) and analysed each of these symptoms separately.

Recovery from the common cold was rated by both the investigator and the patient using the Integrative Medicine Outcomes scale (IMOS) (Steinsbekk et al., 1999). The patient’s satisfaction with treatment was assessed using the Integrative Medicine Patient Satisfaction Scale (IMPSS) (Steinsbekk et al., 1999). Additionally, the number of days off work or school and the use of paracetamol were documented. Further methodological details on the outcomes of this clinical trial are described elsewhere (Keck et al., 2015; Keck et al., 2018).

### Molecular Diagnostic Assays

At baseline, molecular testing for viruses was performed by taking a nasopharyngeal swab from both nostrils with a sterile brush. Total nucleic acid (NA), i.e., deoxyribonucleic acid (DNA) and ribonucleic acid (RNA), was isolated from the samples by means of the QIAamp nucleic acid isolation kit (Qiagen, Hilden, Germany). The amount of isolated nucleic acid was measured by absorbance. For viral testing, an aliquot was applied to the xTAG® Respiratory Viral Panel (RVP) FAST assay (Abbott, Vienna, Austria) and performed according to the instructions of the manufacturer. Complementary DNA (cDNA) were generated from RNA by reverse transcription (RT) and amplified by polymerase chain reaction (PCR) afterwards within the single step RT-PCR reaction. After RT-PCR, the amplicons were hybridized onto virus species-specific DNA probes immobilized on Luminex beads. Virus species were identified using Luminex technology, with signal measurements of virus-specific hybridization signals specified for the xTAG® RVP FAST assay conducted on a Luminex® 200™ System (Luminex, Austin, TX, United States).

### Statistics

The analysis plan of the original analysis was applied to two additional subgroups of patients. Prior to the analysis, patients with and without positive HCoV NA were identified. Descriptive post-hoc comparisons were performed in NA-positive patients with and without proof of HCoV (HCoV/non-HCoV subset) at baseline. Patients in whom HCoV as well as non-HCoV species had been detected were assigned to the HCoV subset. Within each subset, descriptive summary statistics and 95% confidence intervals (CIs) were computed, and *p*-values were determined for change over time. At least one post-baseline assessment of the analysed outcome was required for eligibility of the analyses of the course of the common cold as documented by the investigators (full analysis set, FAS). In this case, missing data were replaced by carrying the last valid observation forward. No replacement strategy was applied in cases of the analysis of data derived from the patient diaries. Two-sided *p*-values of up to 0.05 were considered to be descriptively significant for statistical tests (Fisher’s exact test and Mann-Whitney-U-test for qualitative and quantitative between-group comparisons, respectively; Wilcoxon signed rank test for within-group comparisons). Data are given as mean ± SD if not stated otherwise.

## Results

### Patients

Between January 2011 and November 2012, 120 patients were enrolled and underwent treatment. Swab samples were available from 119 patients and virus NA were detected in 61 patients (51.3%).

Of the 61 patients, 23 (37.7%) were HCoV-positive and 38 (62.3%) HCoV-negative (Table 1).

**TABLE 1:** Baseline characteristics (number (%) of patients or mean ± SD, by HCoV detection).

Virus NA	HCoV (n = 23)	Non-HCoV (n = 38)
Female	17 (73.9%)	27 (71.1%)
Male	6 (26.1%)	11 (28.9%)
Age (years)	34.2 ± 11.7	37.6 ±12.8
Height (cm)	171.3 ± 9.6	172.2 ± 8.2
Weight (kg)	70.4 ± 11.0	72.5 ± 14.7
Duration of common cold symptoms (hours)	50.1 ± 16.3	50.1 ± 16.8
Never smoker	9 (39.1%)	18 (47.4%)
Former smoker	4 (17.4%)	6 (15.8%)
Current smoker	10 (43.5%)	14 (36.8%)
Number of virus species detected		
1	16 (69.6%)	34 (89.5%)
2	5 (21.7%)	4 (10.5%)
3	2 (8.7%)	0 (0.0%)

In the HCoV subset, 11/23 (47.8%) patients tested positive for HCoV-HKU1, 7/23 (30.4%) for HCoV-OC43, 2/23 (8.7%) for HCoV-NL63, and 4/23 (17.4%) for HCoV-229E. Of these, 7/23 (30.4%) patients of the HCoV subset tested positive for more than one virus: 1 patient for both HCoV-HKU1 and influenza B virus; 1 patient for both HCoV-HKU1 and respiratory syncytial virus (RSV) A; 2 patients for both HCoV-HKU1 and enterovirus/rhinovirus; 1 patient for both HCoV-229E and enterovirus/rhinovirus; 1 patient for HCoV-HKU1, HCoV-OC43, and RSV A; and 1 patient for HCoV-HKU1, enterovirus/rhinovirus, and parainfluenza virus 2.

In the non-HCoV subset, 25/38 (65.8%) patients tested positive for enterovirus/rhinovirus, 4/38 (10.5%) for parainfluenza virus 2, 4/38 (10.5%) for influenza A virus, 2/38 (5.3%) for RSV A, 3/38 (7.9%) for human metapneumovirus (HMPV), 2/38 (5.3%) for RSV B, 1/38 (2.6%) for parainfluenza virus 1, and 1/38 (2.6%) for parainfluenza virus 3. Of these, 4/38 (10.5%) patients tested positive for more than one virus: 1 patient for both enterovirus/rhinovirus and parainfluenza virus 1; 2 patients for both enterovirus/rhinovirus and parainfluenza virus 2; 1 patient for both parainfluenza virus 2 and RSV A.

One patient of the non-HCoV subset withdrew consent prior to the first post-baseline assessment without giving the reason. Therefore, 23 and 37 patients could be analysed for the HCoV and non-HCoV subset, respectively.

There were no statistically significant differences in baseline characteristics between the HCoV and non-HCoV subset concerning gender, age, height, weight, or duration of common cold symptoms prior to trial inclusion (Table 1). All patients but one were Caucasians. Slightly more patients in the HCoV subset were smokers or former smokers compared to the non-HCoV subset (60.9 vs 52.6%).

### Common Cold Symptoms and Further Common Cold-Relevant Complaints

**FIGURE 1:**
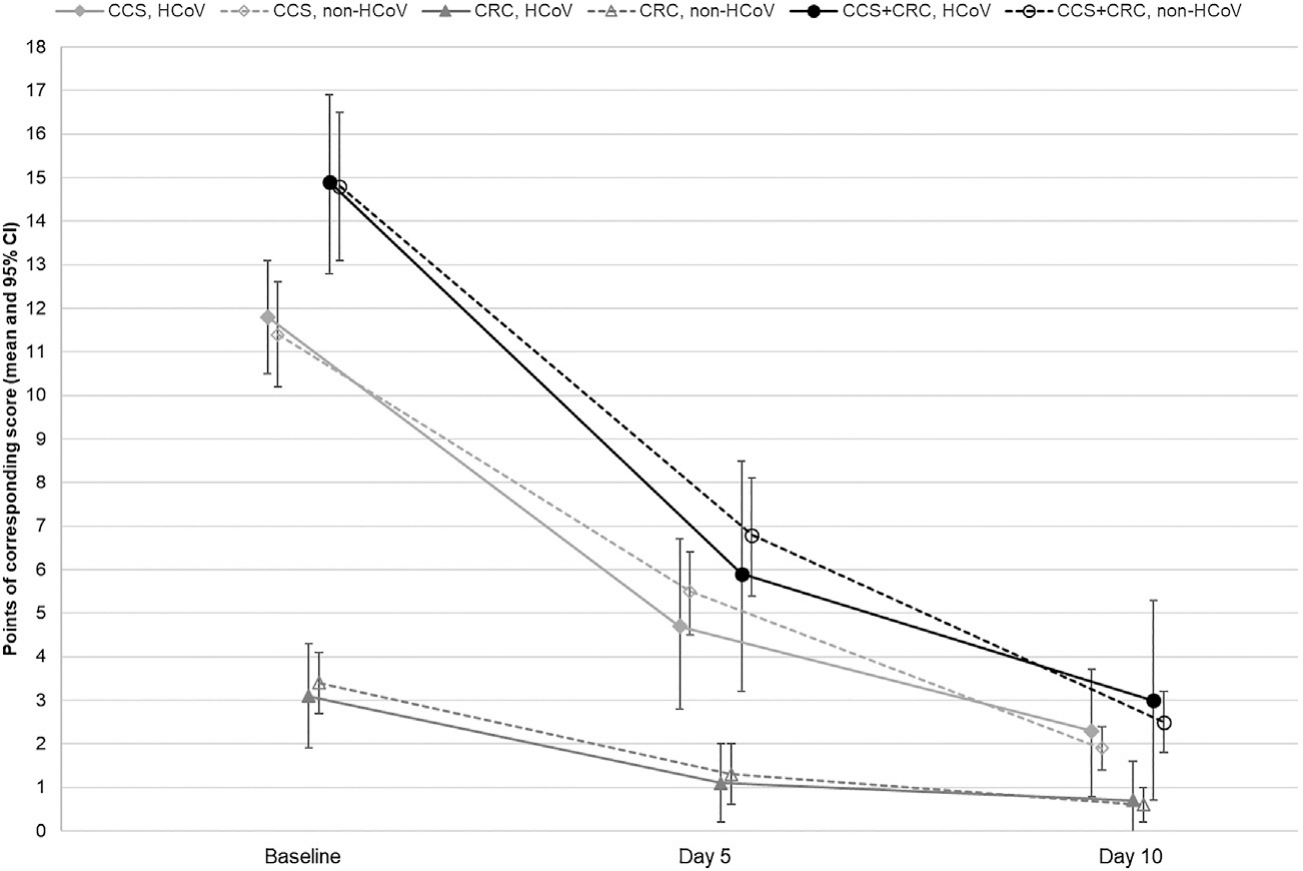
Course of the common cold symptoms (CCS), further common cold relevant complaints (CRC) and overall symptom score (CCS + CRC) in the HCoV and non-HCoV subsets during the trial. CCS, HCoV: CCS investigator rating of patients tested positive for at least one HCoV; CCS, non-HCoV: CCS investigator rating of patients tested positive for viruses other than HCoV; CRC, HCoV: CRC investigator rating of patients tested positive for at least one HCoV; CRC, non-HCoV: CRC investigator rating of patients tested positive for viruses other than HCoV; CCS + CRC, HCoV: CCS + CRC total score subset patients tested positive for at least one HCoV; CCS + CRC, non-HCoV: CCS + CRC total score subset patients tested positive for viruses other than HCoV.

At baseline, patients of the HCoV and non-HCoV subsets showed a similar CCS total score as rated by the investigators (Table 2). After 5 days of treatment with EPs 7630, a somewhat greater improvement in the CCS could be seen in the patients of the HCoV subset compared to non-HCoV patients, but this was no longer the case at the end of the treatment (day 10). At both time points, there were no statistically significant differences between both subsets. Comparable results were observed regarding the time course of the CRC and the overall symptom score (Figure 1; Table 2). Patients of both subsets achieved descriptively significant mean score reductions of ≥50% of baseline values already after 5 days of EPs 7630 treatment and continued to improve until day 10. There were no statistically significant differences between both subsets.

**TABLE 2:** Common cold associated symptoms–baseline total score and change between baseline and subsequent visits (CCS, common cold symptoms; CRC, further common cold relevant complaints; CCS + CRC, overall symptom score; investigator ratings, mean ± SD and Wilcoxon test for change vs. baseline; FAS; *two-sided *p*-value of Wilcoxon signed rank test).

Virus NA		HCoV			Non-HCoV		
		n	Mean ± SD	p*	n	Mean ± SD	p*
CCS	Baseline	23	11.8 ± 3.0		38	11.4 ± 3.6	
	Change, day 5	22	−7.0 ± 5.6	<0.01	37	−5.7 ± 3.5	<0.01
	Change, day 10	23	−9.5 ± 4.3	<0.01	37	−9.4 ± 3.5	<0.01
CRC	Baseline	23	3.1 ± 2.8		38	3.4 ± 2.1	
	Change, day 5	22	−2.0 ± 2.3	<0.01	37	−2.0 ± 1.9	<0.01
	Change, day 10	23	−2.3 ± 2.6	<0.01	37	−2.7 ± 1.6	<0.01
CCS + CRC	Baseline	23	14.9 ± 4.7		38	14.8 ± 5.1	
	Change, day 5	22	−9.0 ± 6.7	<0.01	37	−7.7 ± 4.4	<0.01
	Change, day 10	23	−11.9 ± 5.9	<0.01	37	−12.1 ± 4.4	<0.01

**FIGURE 2:**
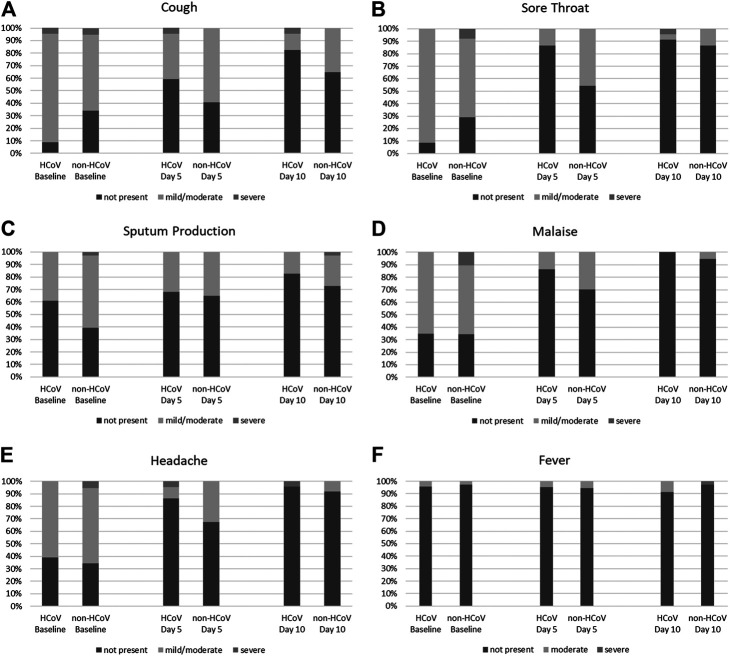
Symptom severity of **(A)** cough, **(B)** sore throat, **(C)** sputum production, **(D)** malaise, **(E)** headache, and **(F)** fever in the HCoV and non-HCoV subsets at baseline, day 5 and day 10 of the trial (% of patients).

Figure 2 depicts the severity of the symptoms fever, cough, malaise, sputum production, headache, and sore throat as rated by the investigator during the trial. Fever was present in only 1 patient of each subset at the start of treatment. In both cases, symptom severity was assessed as moderate. No considerable change was observed during the trial. With respect to the severity of the symptoms cough, malaise, sputum production, headache, and sore throat, patients of both subsets showed comparable improvements; there was a somewhat faster response to the treatment in the HCoV subset. However, these group differences were not statistically significant.

**FIGURE 3:**
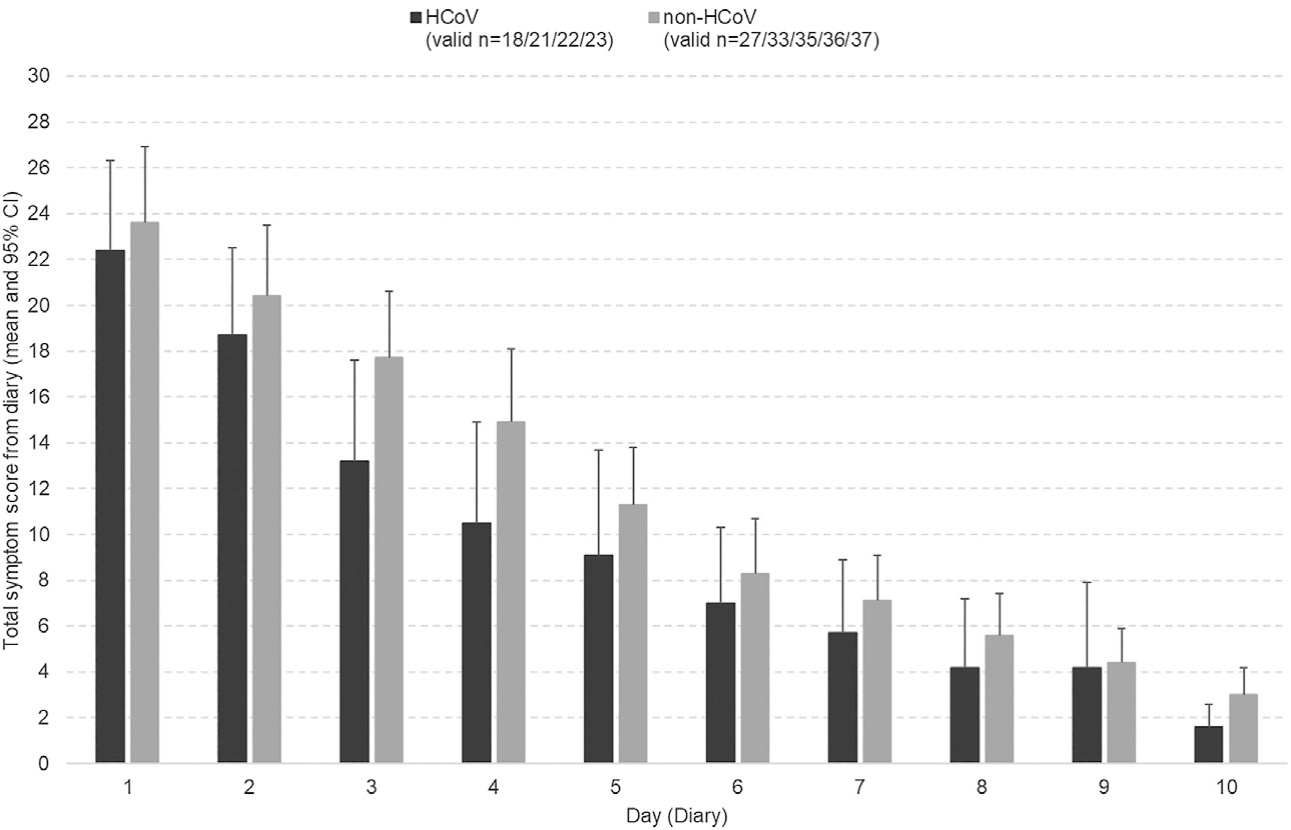
Course of the common cold symptoms (patient diary entries) in the HCoV and non-HCoV subsets during the trial.

The mean total scores of the patients’ self-rating documented in the diaries also showed a continuous improvement in both subsets until treatment end (Figure 3). Again, there was a somewhat faster decrease for the HCoV group until day 5. Nevertheless, no statistically significant differences were seen.

### Recovery From the Disease and Satisfaction With Treatment

**FIGURE 4:**
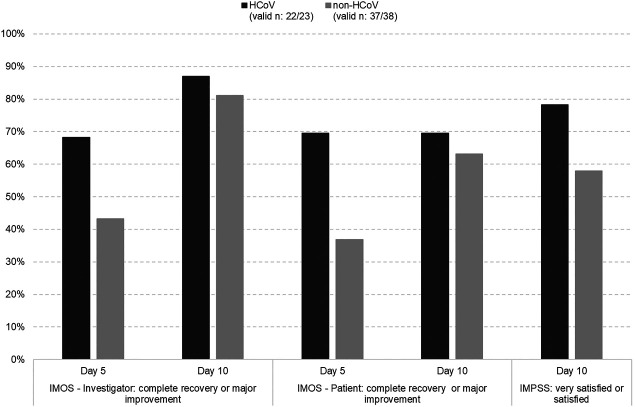
Treatment outcome (IMOS) and satisfaction with treatment (IMPSS)–% of patients with a favourable response in the HCoV and non-HCoV subsets (FAS).

According to the investigators’ IMOS ratings, 68.2% of HCoV patients and 43.2% of non-HCoV patients showed at least major improvements after 5 days of treatment with EPs 7630 (*p* < 0.001, Fisher’s Exact Test). At treatment end, 87.0% of HCoV patients were completely recovered or majorly improved compared to 81.1% of non-HCoV patients (Figure 4).

Moreover, IMPSS indicated a somewhat higher treatment satisfaction in the HCoV cohort, but there were no statistically significant differences between both subsets. A total of 18/23 patients (78.3%) of the HCoV subset were satisfied or very satisfied with treatment. In the non-HCoV subset, 22/38 (57.9%) patients rated satisfaction with treatment accordingly (Figure 4). No patient of the HCoV subset was dissatisfied or very dissatisfied with the treatment compared to 1 patient in the non-HCoV subset.

### Use of Paracetamol

During the trial, paracetamol was taken by a total of 11/61 (18.0%) patients with proof of viral NA. In the HCoV and non-HCoV subset, paracetamol use was reported for 3/23 (13.0%) and 8/38 (21.1%) patients, respectively.

### Inability to Work

With 0.9 ± 2.6 and 1.3 ± 2.8 days, respectively, the mean number of days off work or school/college was comparable for patients in the HCoV subset compared to the non-HCoV subset.

### Tolerability and Safety

A total of 10 adverse events (AEs) were observed in 7 of the 61 patients (11.5%) analysed: 5 AEs were reported by 4 patients (10.5%) in the non-HCoV subset and 5 AEs by 3 patients (13.0%) in the HCoV subset. For the reported 5 AEs in the non-HCoV subset, a causal relationship with the study medication was assessed as unlikely. In the HCoV subset, a causal relationship with the study medication was assessed as unlikely for 2 AEs of 2 patients and as unrelated for 3 AEs in 1 patient. In both subsets, the MedDRA System Organ Class with the largest number of patients affected by an AE was gastrointestinal disorders (non-HCoV: 3 (7.9%) patients; HCoV: 2 (8.7%) patients).

## Discussion

In experimental studies, the proanthocyanidin-rich herbal extract EPs 7630 was shown to display antiviral activity against different viruses including coronaviruses (Kolodziej, 2011; Michaelis et al., 2011; Moyo & Van Staden, 2014). In the context of the ongoing COVID-19 pandemic, it was thus a logical first step to revisit a previously published clinical trial for further evidence on the impact of EPs 7630 on HCoV infections.

In the underlying trial, about half of the patients suffering from the common cold tested positive for at least one of the investigated viruses, and of these, 23 were shown to be infected with various HCoVs (Keck et al., 2015). Our analysis of the clinical manifestations of the common cold found no evidence for major differences between HCoV positive patients and those infected with other viruses.

Moreover, HCoV and non-HCoV subsets had similarly favourable outcomes, with significant improvement of symptoms already after 5 days of treatment and patients having largely recovered after 10 days. There was a not statistically significant trend suggesting superior activity of EPs 7630 in HCoV patients after 5 days of EPs 7630 treatment, which disappeared at the end of treatment at day 10. These results are in line with the total symptom scores calculated from the diary entries. Moreover, the slight but non-significant advantage seen for HCoV patients in improvement of common cold symptoms is reflected in the duration of patients’ inability to work, which was slightly lower in the HCoV subset (0.9 days) compared to the non-HCoV subset (1.3 days).

It remains unresolved whether patients suffering from common cold associated with HCoVs as found in the underlying trial generally recover faster than patients suffering from common cold caused by other viruses or whether they responded faster to treatment with EPs 7630. However, existing studies comparing HCoV infections and viral non-HCoV infections give no hint towards a generally faster recovery from HCoV infections (Lau et al., 2006; Gorse et al., 2009; Gorse et al., 2015). In a prospective cohort-study including more than 4,000 patients hospitalized for ARTI, Lau et al. (Lau et al., 2006) compared clinical characteristics in 629 children aged 6 months to 5 years infected with different viruses. In children, HCoV-HKU1 infections were associated with a shorter duration of fever and hospitalization and HCoV-OC43 infections with a shorter duration compared to most other respiratory viruses. In a prospective cohort study in 2,215 patients with chronic obstructive pulmonary disease (COPD) presenting for influenza vaccination, no differences were observed in terms of symptoms, hospitalization rate or mortality between HCoV and non-HCoV/non-influenza infections (Gorse et al., 2009). A study of HCoV and other virus-associated respiratory illnesses reported by Gorse et al. (Gorse et al., 2015) followed 101 healthy adults aged 18–40 years, a population most similar to the patients included in our trial, for up to 2 years. The study reported 26 cases of ARTI with HCoV and 33 cases with other viruses, but no differences between these groups with regard to the number of symptoms, the symptoms and signs score, or illness severity measured by visual analogue scale. Hence, there is no consistent evidence suggesting a faster recovery from HCoV infections than from non-HCoV viral ARTI. However, available data are limited and further investigations are needed to answer whether there may be differences in disease outcome.

The range of symptoms differed slightly between patients infected with different viruses. Cough and sore throat were reported more frequently in the HCoV group at baseline, and sputum production more frequently in the non-HCoV group. Remission rates at day 5 were highest for sore throat, malaise, and headache. In a meta-analysis of placebo-controlled trials, EPs 7630 treatment effects on day 7 were estimated to be higher for cough and headache than for sputum production (Matthys et al., 2016). Apparently, the somewhat faster recovery of symptom scores in the HCoV group may reflect differences in the nature of symptoms caused by HCoV and non-HCoV infections and the differential impact of EPs 7630 treatment on varying symptoms.

Differences in patient characteristics might have contributed to the somewhat faster recovery in the HCoV group of the underlying trial, as patients in this group were slightly younger. By contrast, the Body Mass Index, the proportion of current smokers and the rate of infection with multiple viruses were higher in the HCoV group. However, these differences were not statistically significant.

The results of our subset analyses are in line with the results obtained for the whole population of the underlying trial (Keck et al., 2015; Keck et al., 2018). Therefore, it is not surprising that patients who tested positive for HCoV NA showed a comparable course of disease under EPs 7630 treatment as patients infected with other viruses.

The exact pharmacological mechanisms underlying these effects are not yet fully understood, but they are most likely attributable to the antiviral activity against different viruses including corona viruses shown for EPs 7630 in experimental studies (Kolodziej, 2011; Michaelis et al., 2011; Moyo & Van Staden, 2014). Moreover, immunomodulatory activities and increased ciliary beat frequency (Kolodziej, 2011; Luna et al., 2011; Theisen & Muller, 2012; Tahan & Yaman, 2013; Helfer et al., 2014; Witte et al., 2015; Roth et al., 2019; Witte et al., 2020) could be beneficial in the context of respiratory viral infections.

Patients suffering from COVID-19 are likely to use herbal medicinal products for early and/or symptomatic management, particularly for cough, pain, or fever (Silveira et al., 2020). Results of our comparative analysis show high improvement rates for cough and sore throat as well as sputum production, headache, and malaise.

It should be noted, however, that co-medication with paracetamol was reported by less than 22% of patients, with no considerable differences between both subsets. This mirrors results from two meta-analyses of randomized, double-blind, placebo-controlled trials showing a reduced use of paracetamol in EPs 7630-treated children suffering from ARTI such as acute bronchitis and acute tonsillopharyngitis (Seifert et al., 2019; Seifert et al., 2021). EPs 7630 has no known direct antipyretic effect. Thus, the authors of the meta-analyses interpreted the fairly low use of paracetamol as an indicator of a favourable disease course in which only a minority of EPs 7630-treated patients developed fever that required antipyretics. Patients may have benefitted from EPs 7630 due to the pharmacological activity such as alleviation of cytokine-induced sickness behaviour and antiviral activity as demonstrated in non-clinical studies (Nöldner & Schötz, 2007; Theisen & Muller, 2012).

The improvement of symptoms observed in both subsets is also in line with results reported from randomised, placebo-controlled clinical trials in patients suffering from the common cold (Lizogub et al., 2007; Riley et al., 2018; Riley et al., 2019). In these trials, the symptoms nasal drainage, sore throat, nasal congestion, sneezing, scratchy throat, hoarseness, cough, headache, muscle aches, and fever were assessed using an observer-rated cold intensity score (CIS). The mean CIS as well as the severity of all individual symptoms decreased at a noticeably higher rate in the EPs 7630 groups compared to the placebo group. This also led to a statistically significant reduction of the primary outcome measure (the sum of symptom intensity differences of the CIS) in these trials (Lizogub et al., 2007; Riley et al., 2018; Riley et al., 2019). Moreover, treatment outcome was significantly better than placebo according to the IMOS (Lizogub et al., 2007; Riley et al., 2018; Riley et al., 2019) and patients’ treatment satisfaction was significantly higher according to the IMPSS (Riley et al., 2018; Riley et al., 2019), which is also in accordance with the results of the present subset analysis. The same applies to the reduced loss of working or study days reported by EPs 7630-treated patients (Lizogub et al., 2007; Riley et al., 2018).

A limitation of the present interventional trial is that it was not intended for demonstrating treatment efficacy but to investigate EPs 7630 in a well-defined, real-world setting. Due to the lack of a control group, effects under treatment must be interpreted cautiously. Nevertheless, the extent of symptom relief was similar to that in double-blind, randomised, controlled clinical trials in adults suffering from the common cold (Lizogub et al., 2007; Riley et al., 2018; Riley et al., 2019), in which EPs 7630 was shown to be significantly superior to placebo. As a second limitation, patients who had both a positive test for HCoV and other viruses were not analysed separately, since patient numbers were too small. It remains unclear if the presence of more than one virus is associated with greater disease severity. Moreover, objective endpoints such as cytokine levels, computed tomography of lung, or quantitative PCR were not recorded, which prevents conclusions on the potential modes of action of EPs 7630.

Our results suggest the hypothesis that EPs 7630 may exhibit beneficial effects in the treatment of HCoV infections and may therefore be promising for early therapy or mild courses of SARS-CoV-2 infections. However, further non-clinical studies and clinical trials are needed to investigate the therapeutic option of proanthocyanidin-rich herbal drug preparations like EPs 7630 in HCoV infections in greater detail.

## Conclusion

Our analyses showed no difference in the disease course between acute respiratory tract infections in EPs 7630-treated patients who tested positive for HCoV or other respiratory viruses. As this trial was conducted before the pandemic, there is currently no evidence from clinical trials for the efficacy of EPs 7630 in patients with SARS-CoV-2 infection. Dedicated non-clinical studies and clinical trials are warranted to further elucidate the potential of EPs 7630 for the early treatment of HCoV infections. Overall, the presence or absence of HCoV in common cold does not seem to have an impact on treatment outcomes.

## Data Availability

The datasets presented in this article are not readily available because raw data cannot be shared both due to ethical reasons and to data protection laws. To the extent permitted by law, the trial data required for validation purposes have already been disclosed in result reports on corresponding databases. All relevant data are within the paper. Requests to access the datasets should be directed to the corresponding author.
